# An Experimental and Simulation Study of the Active Camber Morphing Concept on Airfoils Using Bio-Inspired Structures

**DOI:** 10.3390/biomimetics8020251

**Published:** 2023-06-13

**Authors:** Alexsteven Dharmdas, Arun Y. Patil, Azar Baig, Owais Z. Hosmani, Shridhar N. Mathad, Mallikarjunagouda B. Patil, Raman Kumar, Basavaraj B. Kotturshettar, Islam Md Rizwanul Fattah

**Affiliations:** 1 School of Mechanical Engineering, BVB Campus, KLE Technological University (B. V. Bhoomaraddi College of Engineering and Technology), Vidya Nagar, Hubballi 580031, India; alexdharmdas@gmail.com (A.D.); azharbaig892@gmail.com (A.B.); owaishosmani@gmail.com (O.Z.H.);; 2 Department of Physics, KLE Institute of Technology, Hubballi 580030, India; physicssiddu@gmail.com; 3 Bharat Ratna Prof. CNR Rao Research Centre, Basaveshwar Science College, Bagalkot 587101, India; mallupatil04@gmail.com; 4 University Centre for Research and Development, Department of Mechanical Engineering, Chandigarh University, Mohali 140413, India; raman.me@cumail.in; 5 Centre for Technology in Water and Wastewater (CTWW), School of Civil and Environmental Engineering, Faculty of Engineering and IT, University of Technology Sydney, Ultimo, NSW 2007, Australia

**Keywords:** bio-inspired design, morphing wing, variable camber, aerodynamics, shape adaptation

## Abstract

Birds are capable of morphing their wings across different flight modes and speeds to improve their aerodynamic performance. In light of this, the study aims to investigate a more optimized solution compared to conventional structural wing designs. The design challenges faced by the aviation industry today require innovative techniques to improve flight efficiency and minimize environmental impact. This study focuses on the aeroelastic impact validation of wing trailing edge morphing, which undergoes significant structural changes to enhance performance as per mission requirements. The approach to design-concept, modeling, and construction described in this study is generalizable and requires lightweight and actively deformable structures. The objective of this work is to demonstrate the aerodynamic efficiency of an innovative structural design and trailing edge morphing concept compared to conventional wing-flap configurations. The analysis revealed that the maximum displacement at a 30-degree deflection is 47.45 mm, while the maximum stress is 21 MPa. Considering that the yield strength of ABS material is 41.14 MPa, this kerf morphing structure, with a safety factor of 2.5, can withstand both structural and aerodynamic loads. The analysis results of the flap and morph configurations showed a 27% efficiency improvement, which was confirmed through the convergence criteria in ANSYS CFX.

## 1. Introduction

Today, we are in an era of bio-inspired product designs, the inspiration for which comes from nature, as nature has devastating as well as healing processes built into it. Aircraft systems are one such product derived from nature, especially from birds such as the bald eagle, snow goose, albatross and long eared owl, in order to accomplish a diverse range of adaptations, including in take-off, climbing, cruising, maneuvering and landing [1]. The aerodynamic performance of birds is dynamically maximized as they readjust their wing posture and adapt to different flight conditions [2]. It is generally hypothesized that aircraft which are capable of smooth and continuous shape changing (morphing) would have optimized aerodynamic performance [3]. Morphing wings have gained significant attention in recent days due to concepts such as biomimicry, as well as advancements in electronic components like smart actuators and compliant structures [4]. Morphing concepts have been built based on the theories of weight reduction, minimal energy consumption and enhanced aircraft performance compared to conventional aero-structures [4,5,6,7,8,9,10,11]. The other unique aspect is that surface integrity will not be lost during the flight [4].This problem has been critically examined by FlexSys [12], NASA [13], Boeing, Airbus and other professionals. In small UAVs, polymorphing wings are able to work for chord and camber morphing, Showing the attainment of up to 10% chord extension and ±20% camber morphing modifications [13]. A variable-sweep-wing morphing concept was adopted for four configurations, such as subsonic/supersonic/hypersonic cases [10].

The term morphing coverage a wide range of subjects in the engineering discipline. The scope mainly covers design for optimization, aerodynamic optimization, aero structure optimization and manufacturing process development. Morphing technology encompasses material selection and analysis, structure and solid mechanics developments, smart materials for actuation and conventional actuators for morphing. In a case of morphing wing analysis, authors conducted analytical and experimental modeling, validation, performance and characteristics analysis and aero elasticity analysis [14]. Most of the prior research on aeronautical morphing structures has been on camber-morphing wings and adaptive trailing-edge technologies [15,16,17,18,19,20,21,22,23]. Camber-morphing is a kind of out-of-plane morphing that bends the airfoil’s camber line, modifying the local lift distribution to steer and maneuver the aircraft, essentially turning a low-lift airfoil shape into a higher performance, high-lift airfoil shape. Traditional aircraft use trailing-edge discrete surfaces to adjust the camber of the wing, but camber-morphing surfaces give a seamless shape with no extra gaps, avoiding the huge drag profiles associated with traditional control surfaces. Majid and Jo [14] compared the aerodynamic performance of camber-morphing and conventional airfoils. This study demonstrated the advantages of variable camber-morphing wings over conventional wings, demonstrating their superiority in terms of aerodynamic efficiency, agility and maneuverability. Substantial study has also been undertaken to propose camber-morphing rib designs [10,14,15,16]. Woods and Friswell [22] introduced the fish bone active camber (FishBAC) concept in one of their contributions in 2012. Bishay et al. [19] presented several adaptations of the FishBAC concept, utilizing a shape-memory alloy (SMA) actuated tail structure. Meanwhile, Schlup et al. [24] incorporated this concept into the tail of the MataMorph-2. Jo and Majid [23] recently completed a CFD simulation of a camber-morphing airfoil in transition, demonstrating that the aerodynamic behavior varies linearly as the camber rate rises. The design of the skin is a significant difficulty in wing morphing. A morphing skin is ideally a continuous surface that can adapt to the contour of the morphing body while remaining structurally robust and sturdy enough to withstand aerodynamic stresses. This necessitates that the skin meet contradictory structural criteria, such as low in-plane stiffness and strong out-of-plane bending stiffness [19]. Aeroelastic materials that can withstand significant strain while retaining a linear elastic profile are anticipated to be perfect for morphing aircraft skin and surfaces [20]. Ahmad and Ajaj [25] investigated the multiaxial mechanical properties of a latex skin for morphing wing applications. Thill et al. [26] provided a thorough study of numerous flexible skins and unique material ideas, focusing on the usage of segmented and corrugated structures, reinforced elastomers and flexible matrix composite tubes. Rediniotis et al. [27] took a different approach, presenting a bio-inspired morphing skin with sliding segmented stiff panels for a hydrofoil, which shown encouraging results for out-of-plane camber-morphing.

The NACA2410 outcomes are benchmarked and compared to analytical ones while adjusting AoA. The SST k-turbulence model with transition and curvature correction was used. It should be noted that this model is less sensitive to flow transitions at the leading edge of the airfoil. The NACA8410 airfoils are also computed using the benchmarked ANSYS FLUENT configuration. Finally, the behavior of a camber morphing wing in transition was simulated using a variable camber wing with end configurations matching the geometric profile of the NACA2410 airfoil on one side and the NACA8410 airfoil on the other [23]. Currently, as computer capacity grows, computational fluid dynamics (CFD) is gaining relevance in aircraft design and analysis. This high-fidelity tool has offered precise insights into the flow field, facilitating understanding of aerodynamic noise causes and functioning as a potent supplement to airfoil performance investigation and optimization, avoiding time- and cost-intensive premature model testing. Rumsey and Ying [28] compiled a large number of research papers on the flow calculation of high-lift multi-element designs. RANS approaches have been proven to give good accuracy for a range of 2-D and 3-D experimental scenarios including surface pressures, skin friction, lift and drag, and have therefore become the current most frequent methodology. As a result, there has been significant improvement in noise numerical modelling, where CFD computations are related to an acoustic prediction model using Lighthill’s acoustic analogy [29], which has been applied in various publications for flap-side edge far-field noise predictions [30]. As a result, the combination of CFD and computational aeroacoustics (CAA), as well as their increasing predictive reliability, may become a reliable complement to guide experimental testing towards a more efficient line of research and more promising implementations for (1) improving aerodynamic capabilities and (2) reducing airframe noise.

The predominant focus of previous research on morphing technology’s computational performance has been on aerodynamic studies of specific morphing configurations and their associated processes [31,32], as well as on the deformation and power consumption of morphing designs. The primary objective of this study is to quantify the benefits of morphing wings in terms of drag reduction by comparing various camber morphing rates with different configurations of conventional wings created for different flap deflection angles. The study uses overlaid shapes of the NACA0012 and NACA4412 airfoils, which differ only in their camber while maintaining constant thickness along their chord. The transformation of one airfoil shape to another is achieved through morphing the trailing edge (TE) and leading edge (LE), while the middle section remains unaltered. To facilitate future skin design applications, five lattice structures were chosen as the basis for design optimization and modification, namely (1) honeycomb, (2) auxetic, (3) chiral, (4) zero-ratio Poisson’s honeycomb and (5) square lattices. Each lattice was analyzed using camber adjustments of 1% and 6%, but all models failed the structural analysis due to material elasticity limitations. However, it should be noted that the creation of solitary stress conditions poses a challenge for this finite element method (FEM) investigation, which is reflected in the varying results obtained.

Morphing the geometry of an aircraft’s wing during flight allows for enhanced flight performance across a broader range of flight conditions. Among various methods, camber morphing has emerged as a highly effective and efficient approach for achieving optimal efficiency throughout the entirety of a flight mission [31]. The conventional control systems such as flaps and ailerons, while capable of altering airflow, introduce discontinuities on the wing surface, leading to aerodynamic losses. Furthermore, these systems are inefficient in terms of aerodynamic performance. By employing a continuous flexible skin that encompasses ailerons and flaps, the noise generated by conventional wings can be significantly reduced. However, abrupt changes in camber create a notable rise in induced drag along the airfoil, particularly at higher lift coefficients. Additionally, such changes promote early flow separation, thereby limiting the maximum lift coefficient. Nevertheless, conventional wing-flap configurations fail to provide the optimal solution for overall aircraft performance due to the varying lift requirements in different flight conditions.

NASA’s Morphing Aircraft Program began in 1998 with the aim of developing shape-changing morphing structure using adaptive materials, micro control systems, and bio-inspired material technologies in order to improve the aircraft’s aerodynamic efficiency. The Morphing Aircraft Structure Program at DARPA has been working on large-scale projects to build morphing flight vehicles that can change shape drastically while in flight. In 2002, NextGen Aeronautics, Lockheed Martin and Raytheon Missile Systems sponsored this program [2]. Woods et al. [32] proposed the fishbone active camber wing concept. The fish bone active camber (FishBAC) idea is based on a flexible skeletal structure inspired by fish anatomy. Wind tunnel research revealed that, using the FishBAC morphing structure, a substantial improvement in the lift-to-drag ratio of 20–25% was achieved across a range of angles of attack as compared to the flapped airfoil. One possible solution is to build a flexible structure that changes its geometry seamlessly, with the capability of morphing an aircraft’s wing shape according to the flight mode without compromising the wing surface integrity. The amount of camber in an airfoil has a major influence on the force generation under fluid flow. Structures with variable camber often take advantage of this fact in fluid dynamic systems. Camber variation is used to produce extremely high lift coefficients during take-off and landing [32].

The primary objective of the present study is to design, develop, and validate a morphing trailing edge concept, structure, and mechanism. The study also aims to examine the aerodynamic characteristics of a morphing wing in order to achieve optimal aerodynamic efficiency while simultaneously preserving the maximum lift coefficient. The experimental model employed in this research comprises NACA 0012 airfoil ribs with a wingspan of 250 mm and a mean aerodynamic chord of 200 mm.

In this approach, the trailing edge of the airfoil structure is attached with a rotary degree of freedom. To generate torque on the flap, an actuation system is installed. Rotating it at different angles changes the effective camber of the airfoil and varies the amount of lift produced. The goal of this study is to investigate the aerodynamic characteristics of morphing wings for improved aircraft performance.

## 2. Kerf Bending Active Camber Concept

The concept of kerf bending active camber is fundamentally inspired by the locomotive pattern of snakes [33]. The airfoil features a square wave design to provide flexibility to the structure, thereby achieving smooth bending at the trailing edge [34]. Snakes move in horizontal body waves, and altering the amplitude and phase of these waves enables them to crawl more efficiently. The process of creating slots on a part to facilitate bending is known as kerf bending. To obtain a smooth curve, slots should be placed close to one another and equally spaced. Thinner and more closely spaced slots result in a smooth curve and greater flexibility, while thicker and wider slots produce sharper bends at the slots. The kerf bending calculator can be used to determine the appropriate thickness for a given radius. When the part is bent, the inner edges of the slots touch, resulting in a smooth curve. Over-kerfing may cause cracks to propagate in the part [35].

### Advantages of Kerf Bending

The advantages of kerf bending include its ability to generate single and double curvature surfaces, its ease and speed of use, and its environmentally friendly nature as it does not require molds [36]. Furthermore, it is applicable to a variety of materials such as wood, plexiglass and metal, and can achieve a wide range of shapes.

**Figure 1:**
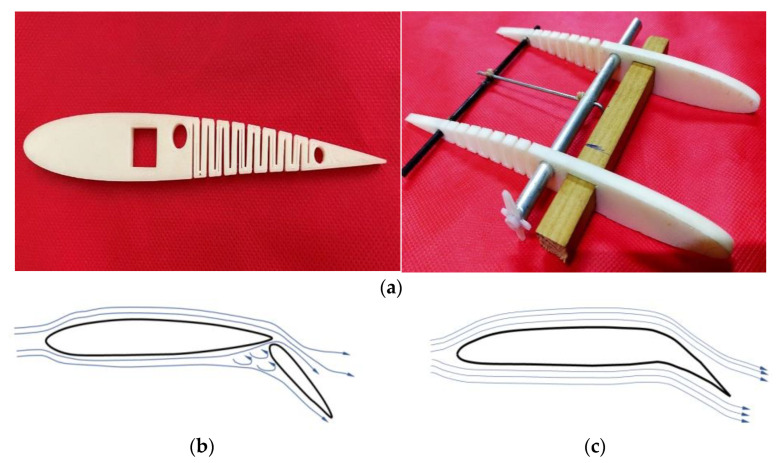
(**a**) Internal morphing structure using kerf rib concept. (**b**) Flow around wing flap configuration. (**c**) Flow around morph configuration.

**Figure 2:**
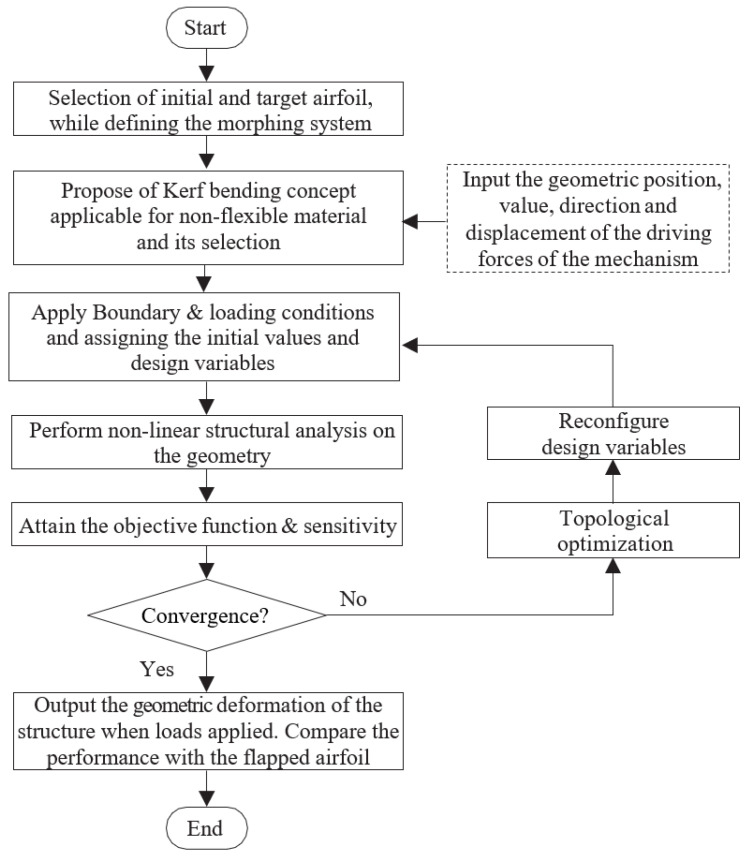
Design flowchart.

In this research, the kerf bending technique is employed to bend a rib, with a focus on ABS material despite the availability of many highly flexible materials. The rib, as shown in Figure 1 and Figure 2, has the kerf concept applied alternatively on its top and bottom surfaces to facilitate smooth bending in both directions. While real-world problems are typically non-linear, this work does not focus on structural analysis behavior but rather on an aerodynamic approach using CFD. There are many ongoing studies focused on addressing the issue of man-made material aspects, although cost aspects remain difficult to estimate at this point.

## 3. Detailed Design of Morphing (Kerf Bending) Structure

**Figure 3:**
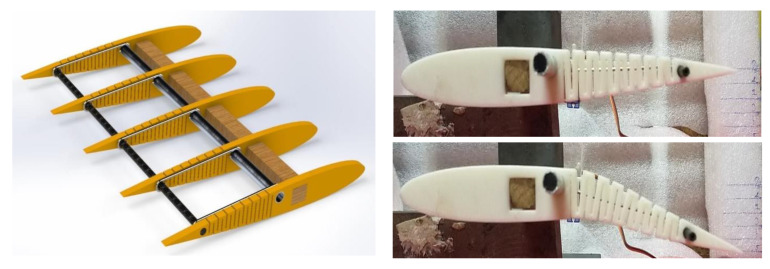
Detailed design of internal morphing structure and mechanism.

**Figure 4:**
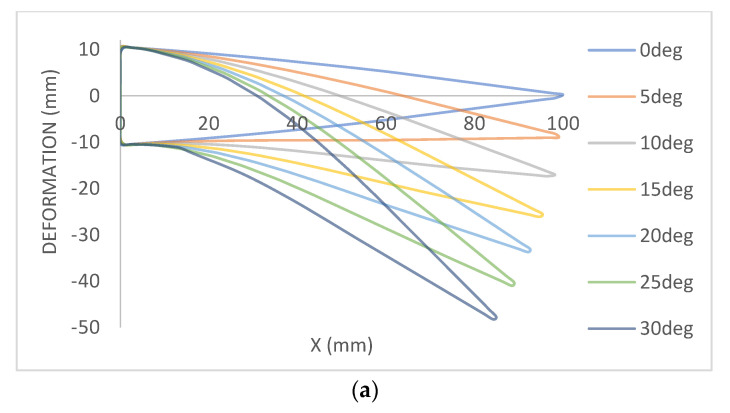

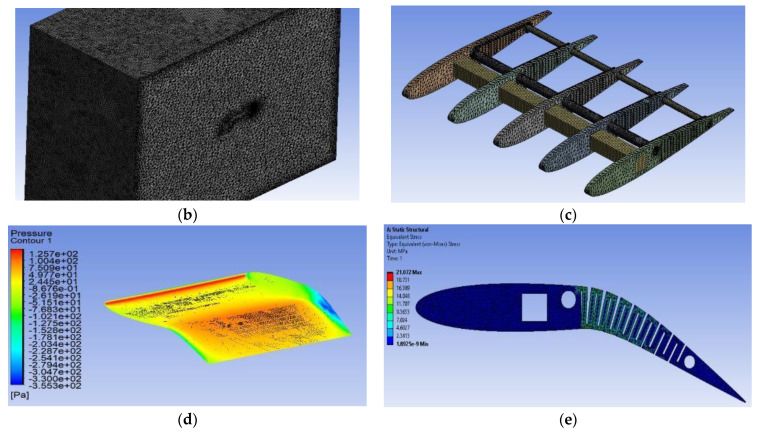
(**a**) Deformation of airfoil under different morphed angles. (**b**) Mesh with 582,434 nodes & 3,211,256 elements. (**c**) Mesh with 200,950 nodes & 80,432 elements. (**d**) Pressure contour at 30 deg deflection. (**e**) von Mises stress.

The detailed design of internal morphing structure and mechanism is as shown in Figure 3. A novel morphing structure has been developed and prototyped, revealing that a symmetric airfoil has superior precision in replicating other airfoils’ properties, characteristic curves and nature, as illustrated in Figure 4a. Conversely, the morphing performance of flat-bottom and cambered airfoils was less precise, as they were incapable of transforming into a symmetric airfoil. Due to factors such as the required velocity to generate lift based on flight regimes and the minimum thickness required for spar insertion to enable morphing for varying camber, the NACA 0012 airfoil was selected based on manufacturing technique and design suitability [37]. The design of this structure aims to achieve maximum bending deflection for rigid materials, thereby enabling camber deflection at a lower energy cost. This structure can bend within a range of −20 to +30 degrees, with increasing slot thickness resulting in greater deflection. Numerous iterations were conducted, and only half of the chord was considered for morphing to obtain the necessary deflection. This deflection is exclusively implemented at the trailing edge of the airfoil to facilitate morphing.

Figure 3 depicts the overall structure of the mechanism, which is 3D printed with a linear pattern and filled with 100% fill. This mechanism is actuated with the help of aluminum rod, by which rotary motion is converted into linear motion with one end of the rod connected to a servo motor placed at the root of the wing and other end connected to the trailing edge spar as shown. This allows us to obtain the targeted deflection of +30 degrees. The deformation curves of 7 deflection angles, with increments of +5 degrees from 0 to +30 degrees, are shown in Figure 4a.

## 4. Static Structural Analysis

Figure 4b,c shows the hybrid mesh used for CFD analysis and structural analysis. The advantage of hybrid mesh [38,39,40] is that it automatically decides the structured mesh [41,42] and unstructured mesh for the normal area and critical area, respectively.

Ansys Fluent was used for CFD analysis. Inlet, outlet and wall were the boundary condition. The cruise velocity was considered at the inlet, i.e., 15 m/s, and density of air was taken as 1.223 kg/m^3^. In this case, the Spalart–Allmaras turbulence model was considered for the viscous case. The maximum pressure generated on the wing was to be 125 Pa, as shown in Figure 4d. This pressure load is applied on the structure in static structural as pressure load. When a pressure load is applied on the structure, the deformation is very small. The maximum displacement is 0.0814 mm, and the stress is only 5 MPa. When considering the wing deflection in the transition from 0 degrees to 30 degrees, the deformation of the structure is shown Figure 4e.

In this case, the maximum displacement at 30 degrees deflection is 47.45 mm, and the maximum stress is 21 MPa. The yield strength of the ABS material is 41.14 MPa. This kerf morphing structure with a safety factor of 2.5 can withstand structural and aerodynamic loads.

## 5. Aerodynamic Performance Analysis

To gain a deeper understanding of the significance of aerodynamic parameters for the NACA0012 airfoil, extensive iterations were carried out using XFLR5 at different morphing angles. The resulting aerodynamic characteristics were subsequently compared to those of a conventional wing-flap configuration. This comparative analysis allows for a comprehensive assessment of the aerodynamic performance of the NACA0012 airfoil relative to a conventional wing-flap configuration, shedding light on the importance of these parameters in the context of aerodynamic efficiency and effectiveness.

### 5.1. Characteristics of NACA 0012 for Different Morph Angles

**Figure 5:**
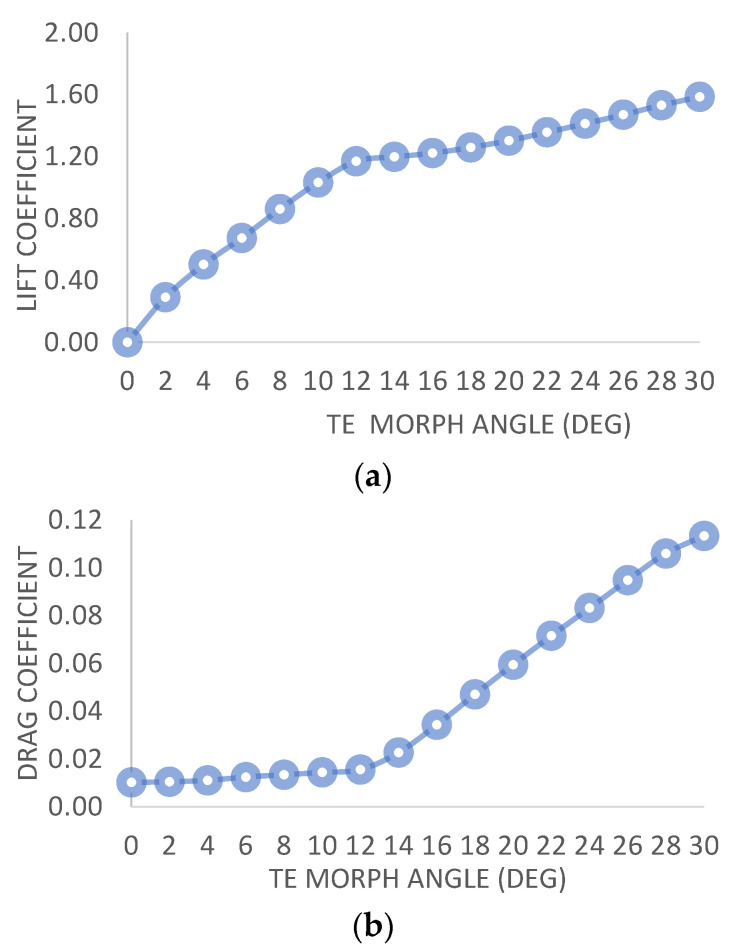

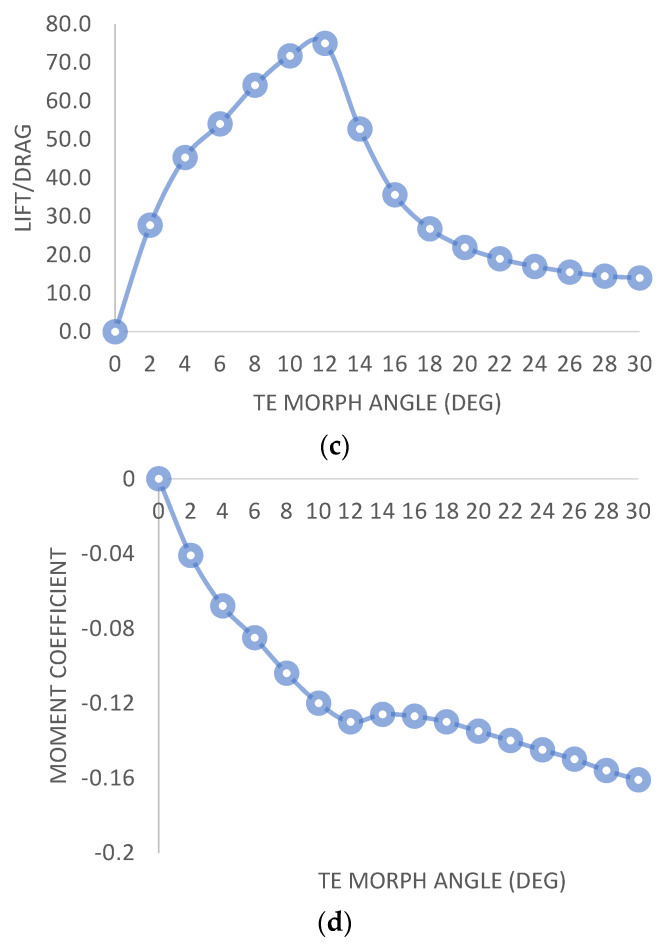
(**a**) C_l_ vs. TE morph angle. (**b**) C_d_ vs. TE morph angle. (**c**) C_l_/C_d_ vs. TE morph angle. (**d**) C_m_ vs. TE morph angle.

The findings indicate that there is a linear relationship between the lift coefficient (Cl) and the morphing angle deflection, with an increase in Cl as the angle deflection rises from 0 to 12 degrees. However, beyond 12 degrees, the rate of change in Cl becomes less significant while the drag coefficient (Cd) increases (Figure 5a,b). Initially, for a NACA0012 symmetric airfoil, the Cl/Cd ratio is 0. However, there is a significant and rapid increase in the Cl/Cd ratio from 0 to 12 degrees of morphing angle deflection. Subsequently, further increases in the deflection angle result in stalling, leading to a sudden decrease in the Cl/Cd ratio (Figure 5c). Moreover, there is a linear decrease in the pitching moment with an increase in the morphing angle until the critical angle is 12 degrees. Beyond this critical angle, the pitching moment continues to follow the same linear pattern (Figure 5d).

**Figure 6:**
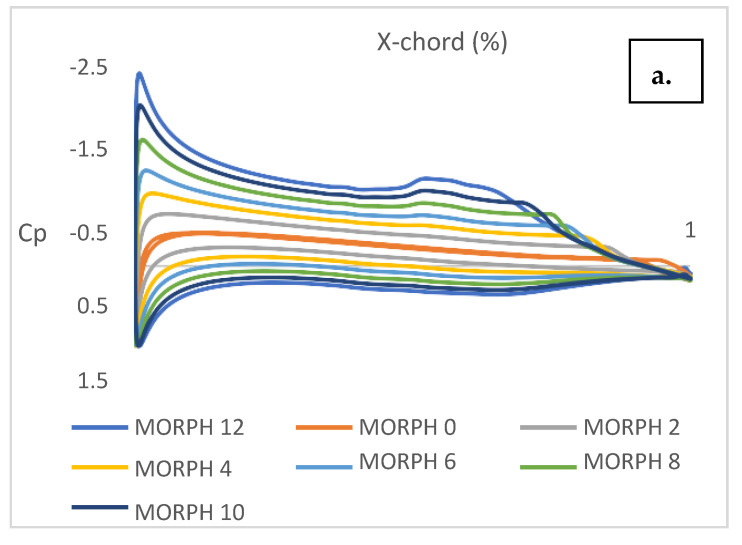

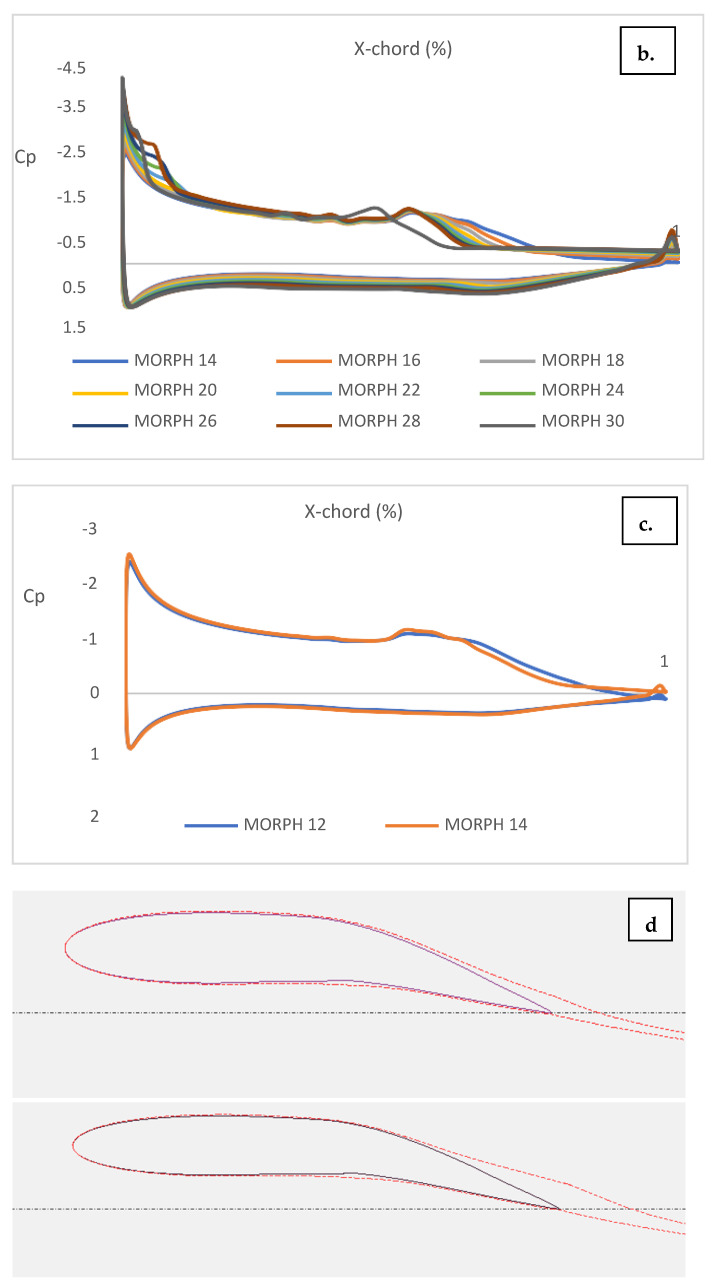
(**a**) Cp of morph angle 0 to 12 degrees. (**b**) Cp of morph angle 14 to 30 degree. (**c**) Transition of flow separation. (**d**) Flow separation.

The observed non-linearity in the lift and drag coefficients with changes in morphing angles can be attributed to flow separation, as evidenced in Figure 6a. Notably, there is a significant change in the pressure coefficient (Cp) distribution between the range of 0 to 12 degrees and 14 to 30 degrees, as depicted in Figure 6b,c. However, there is no significant change in the Cp distribution along the airfoil, indicating that only the aft portion contributes to the increase in lift. Conversely, at the rear part, flow separation becomes more pronounced with increased deflection angle, resulting in higher drag. Figure 6c clearly shows the flow separation during the transition from 12 to 14 degrees of morphing angle. Examining Figure 6d, it is evident that flow separation initiates at a morph angle of 14 degrees. Therefore, the morphing mechanism exhibits excellent performance and maintains a linear pattern from 0 to 12 degrees. However, the efficiency gradually diminishes at higher morph angles, indicating a decrease in overall performance.

### 5.2. Characteristics of NACA 0012 for Different Flap Angles

**Figure 7:**
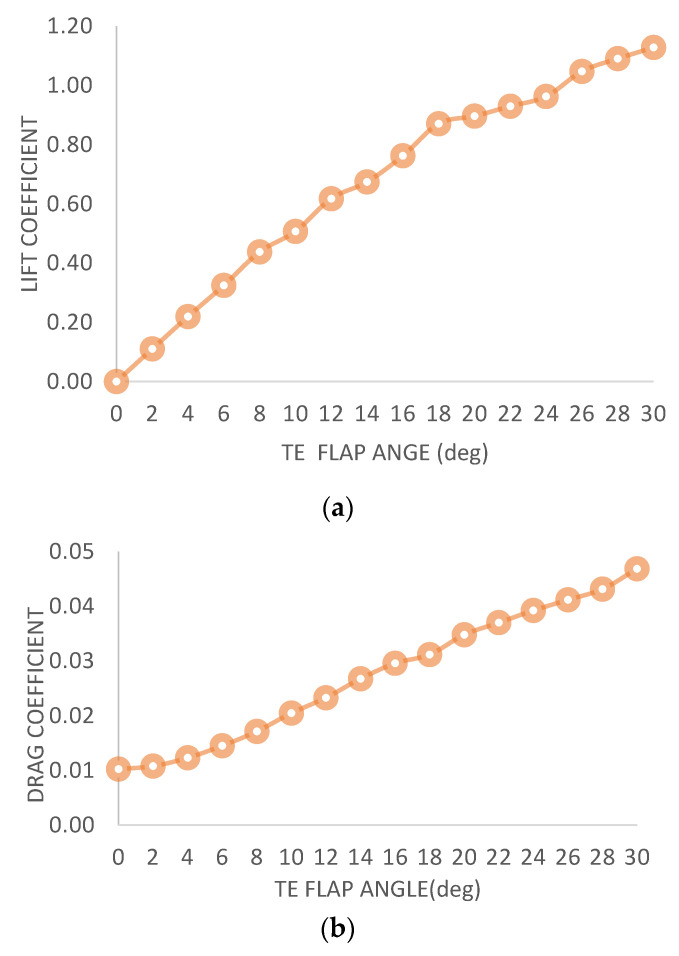

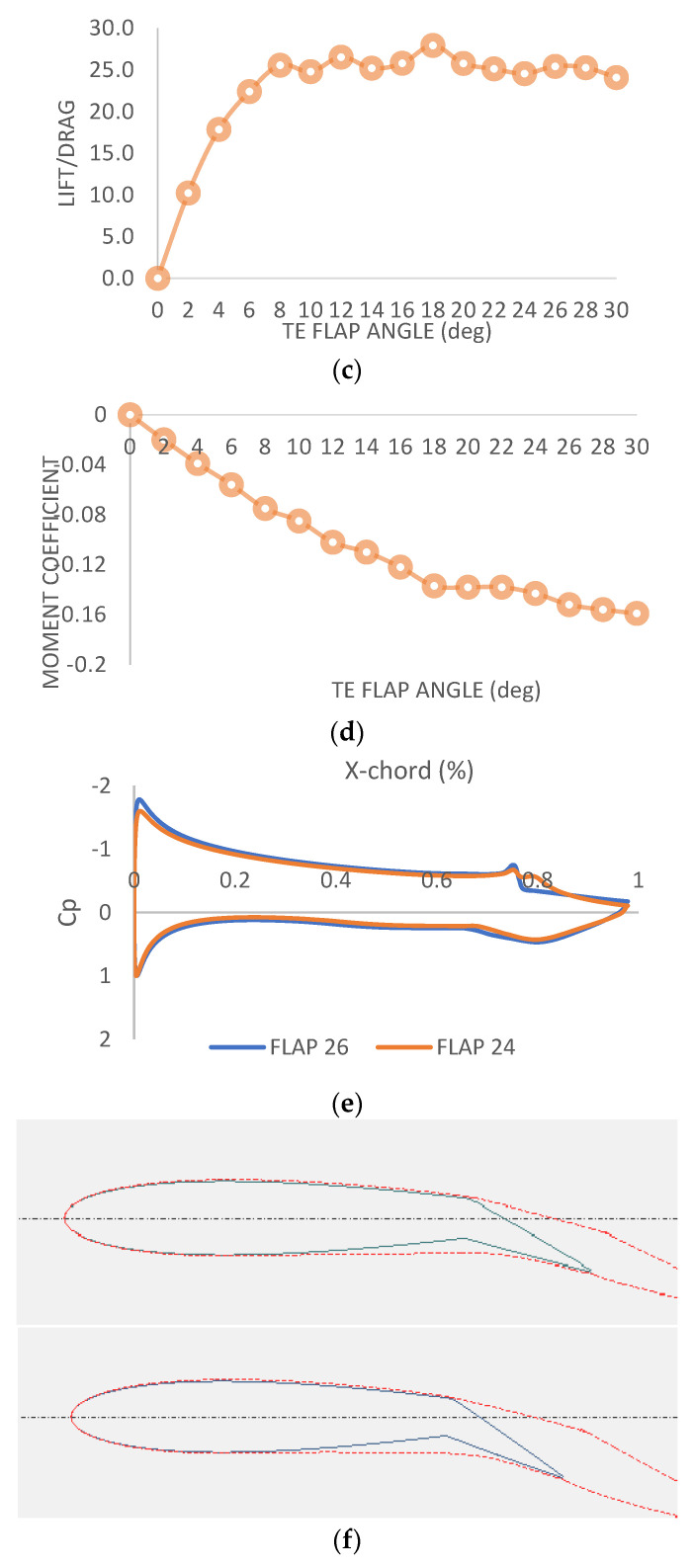
(**a**) C_l_ vs. TE flap angle. (**b**) C_d_ vs. TE flap angle (**c**)**.** C_l_/C_d_ vs. TE flap angle. (**d**) C_m_ vs. TE morph angle. (**e**) Transition of flow separation. (**f**) Flow separation.

To compare the performance of the aforementioned experiment, comparable iterations were performed on a wing-flap configuration. The resulting graphs indicate that the lift coefficient and drag coefficient exhibit nearly linear behavior. The iterations were conducted using XFLR5, and the maximum converged value was observed at a 46-degree flap angle. Figure 7a–f shows that the Cl/Cd ratio has three stages: an initial stage from 0 to 8 degrees in which it linearly increases, a second stage in which it remains stagnant from 8 to 22 degrees, and a final stage in which it decreases sharply beyond 22 degrees.

Observations revealed that the symmetric airfoil is capable of more precise morphing and replication of the properties, characteristic curves, and nature of other airfoils, as shown in Figure 4a. In contrast, the flat bottom and cambered airfoils were less precise as they were unable to morph into a symmetric airfoil. Considering parameters such as the velocity required to generate the required lift according to flight regimes and the minimum thickness required for spar insertion to make the morphing mechanism work for varying camber, the NACA 0012 airfoil was selected based on its manufacturing technique and design suitability. The NACA 0012 airfoil is commonly used in aircraft with an angle of incidence that will generate lift at zero angle of attack [43]. After comparing its performance with other airfoils, including morphing capabilities, it was found that the NACA 0012 airfoil performed well.

**Figure 8:**
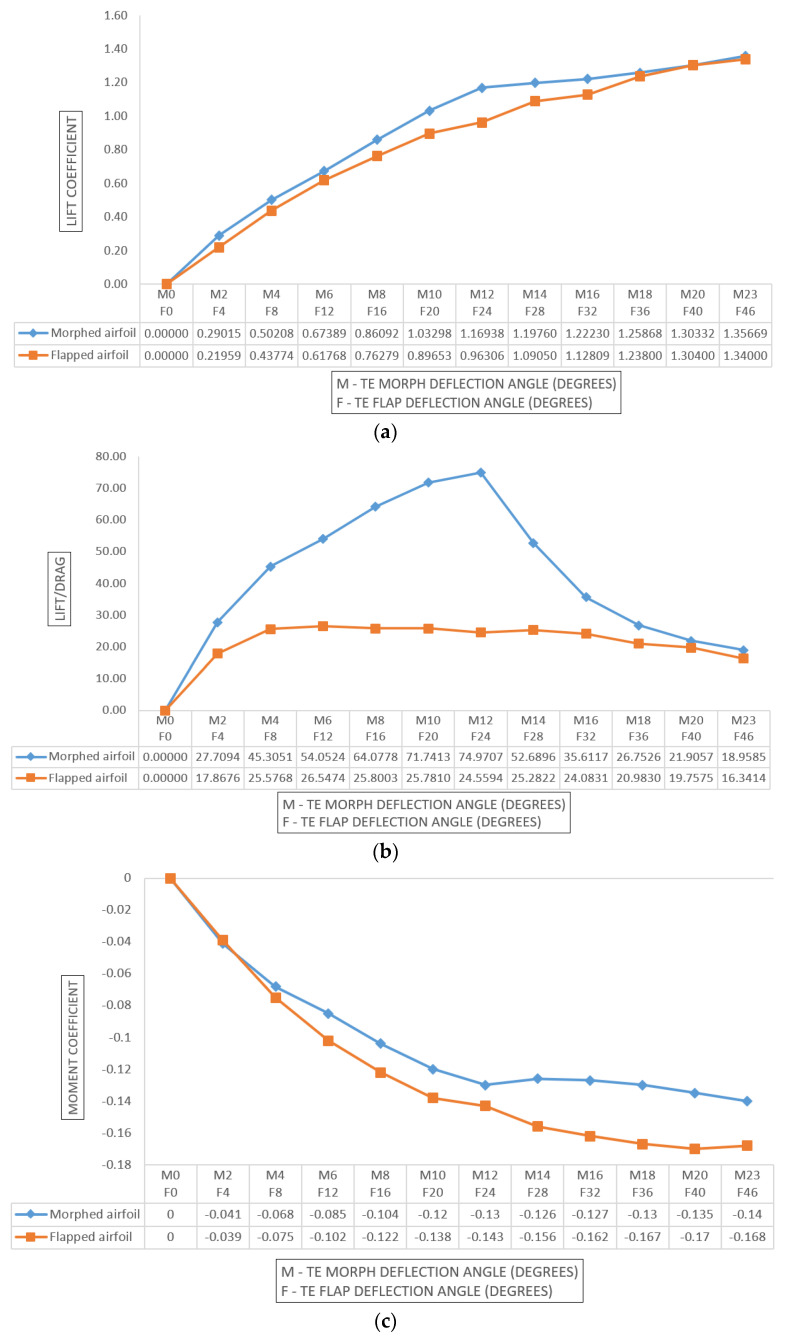
(**a**) Lift coefficient vs. TE morph and flap angle. (**b**) Lift/drag vs. TE morph and flap angle. (**c**) Moment coefficient vs. TE morph and flap angle.

From Figure 8a–c, it is observed that the flow separation initiates at 22 degrees of flap angle, but the separation is noted at 0.75% of the chord, i.e., where the flap is hinged. Hence, it does not affect the linearity of the graph, even after flow separation taking place.

From Table 1, it is understood that Characteristic nature of curve (slope) will discuss on the various parameters. In this particular case study, the lift coefficient (Cl) and drag coefficient (Cd) were calculated for both the morphed and flapped versions of a 250 mm × 200 mm wing. Refer to Table 2 for the data. The efficiency was evaluated in terms of the improvement in Cl/Cd of the morphed wing as compared to the flapped wing, which was found to be approximately 27% after calculation. The authors aimed to demonstrate the concept and claims using a minimal amount of data, presenting the study in a simplified way while conveying the facts in terms of proportionality. For example, the claim that the morphed wing reduces fuel consumption is supported by the fact that the lift-to-drag ratio (L/D) is inversely proportional to fuel/power consumption [44].

Aircraft wing camber is increased with the use of slotted flaps, resulting in a rising curve from the leading edge to the trailing edge. This increased camber leads to an increase in wing surface area, with high pressure escaping through the opening. As a result, the formation of turbulent air behind the wing is delayed, particularly at high angles of attack [45]. After considering all types of flaps, a slotted flap was chosen for this study, as it adds energy to the wing’s boundary layer, delays airflow separation and produces less drag, thereby providing additional lift without excessive drag.

The authors aim to conduct a theoretical analysis of the aerodynamic features of the morphed wing as opposed to the flapped wing. By studying the 2D features, the performance and behavior of the morphed wing can be comprehended at low aspect ratios, such as the dimension of 250 mm × 200 mm, which is considered a worst-case scenario. Furthermore, the results presented in this paper demonstrate that even at higher aspect ratios, the use of a morphed wing can lead to better performance compared to any traditional wing design. The authors aim to validate this claim.

### 5.3. Performance Analysis: Comparison of Flapped and Morphed Airfoil

In order to compare the performance of flapped and morphed airfoil, various case studies were performed—for instance, X angle of flap-deflection generated Y valve of lift-coefficient—in order to understand the significance of morph angle deflection in terms of lift coefficient. For the same lift coefficient, required flap and morph angle deflection were matched and tabulated. It was found that the required morph angle was 0.5% of X angle of flap deflection. Thereafter, matching the lift coefficient for different flap and morph angle deflections, Cl/Cd was compared in order to understand the performance.

**Figure 9:**
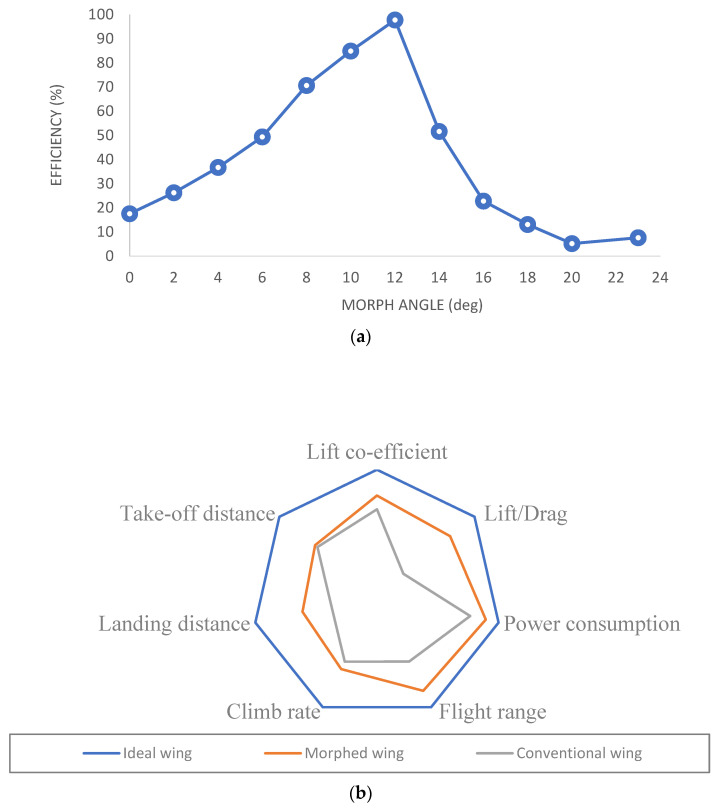
(**a**) Efficiency of 2D morphed airfoil compared to flapped airfoil. (**b**) Graphical representation of Pugh chart in comparison of conventional wing with morphed wing.

**Figure 10:**
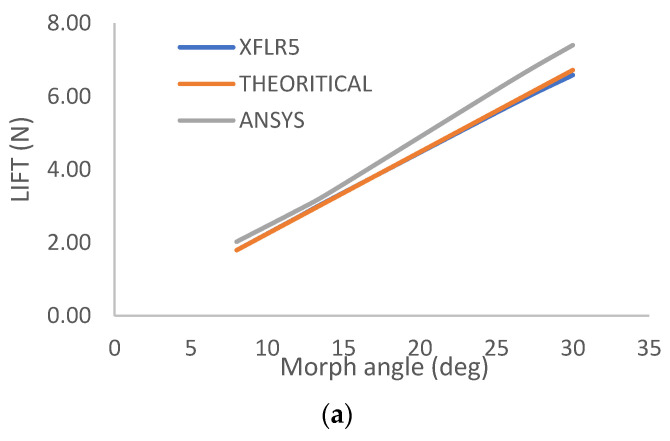

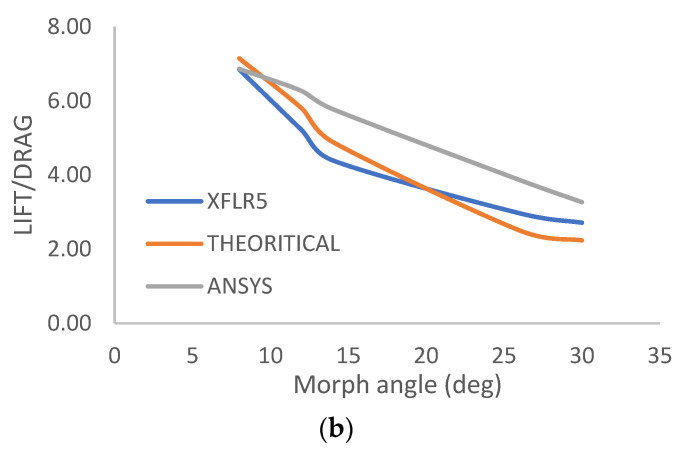
(**a**) Lift vs. TE morph angle. (**b**) L/D vs. TE morph angle.

Upon comparing the performance of morphed and flapped airfoils, it was observed that the rate of lift coefficient increase per degree of morph angle was almost twice that of a flapped airfoil. Additionally, the drag coefficient per degree increment in morph angle was significantly lower than that of a flapped airfoil. Furthermore, the negative pitching of morphed airfoils was found to be less than that of flapped airfoils. The efficiency of a 2D morphed airfoil compared to a flapped airfoil and a graphical representation of the Pugh chart comparing a conventional wing with a morphed wing are discussed in Figure 9a,b. As demonstrated in Figure 10a,b, the overall C/D of the morphed airfoil is consistently higher than that of the flapped airfoil, regardless of the lift coefficient.

A case study was conducted on a wing with a wingspan of 250 mm and a mean aerodynamic chord of 200 mm. This wing was fabricated to test the mechanism and conduct a 3D analysis using computational fluid dynamics (CFD). The validation of this study is planned to be conducted through wind tunnel testing in the future. The lift generated by the wing was measured to be 6 N, which is similar to that generated by indoor mini-RC planes. It was noted that if scaled up, the performance of the wing would remain the same with respect to the comparison of flap and morph and could be used for conventional planes.

CFD analysis was performed in Ansys Fluent and was verified using XFLR5 data and theoretical results. An overall efficiency increase of up to 27.489% in terms of lift-to-drag ratio was observed when compared to a conventional fixed wing-flap configuration.

The work represents an inception or preliminary study of a bio-inspired based model. The entire work was first initiated virtually to understand the feasibility aspect of it. The results have been validated and justified through convergence theory, a well-known method of validating outcomes. The scope of future work includes all experimental studies with detailed comparative analysis of analytical and experimental results. Therefore, initial level studies require simulation with all parameters considered as per real-time conditions.

### 5.4. Fatigue Analysis

The comparative study of various kinds of materials for the fatigue design life cycle was tabulated for active camber design. The results show fatigue life, the factor of safety, damage and bi-axiality indication for various materials, such as acro butadiene styrene (ABS), poly vinyl chloride (PVC), and structural steel, for comparative study. The details of the same were discussed in the following content.

#### 5.4.1. ABS

**Figure 11:**
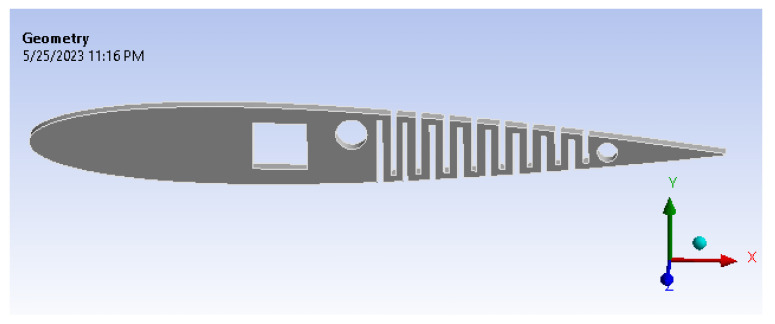
The geometry of the active camber.

ABS has been a top choice for materials when it comes to additive manufacturing, such as 3D printing. Figure 11 shows the geometrical image of the active camber.

**Figure 12:**
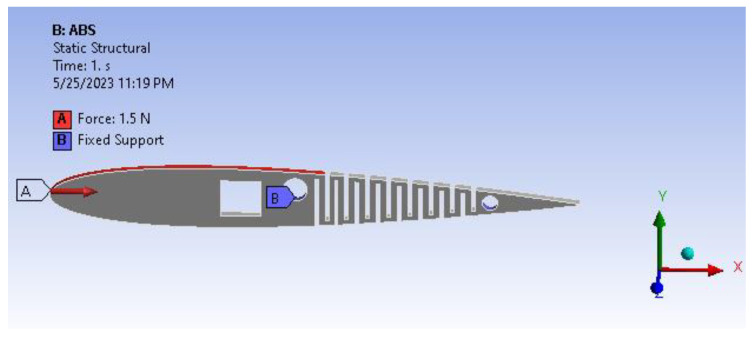
Geometry of the active camber.

For actuation, we used an SG-90 servo motor with a torque of 1.8 kg-cm; to achieve the desired 30 degrees of deflection, we need a minimum force of 1.5 N, as shown in Figure 12.

**Figure 13:**
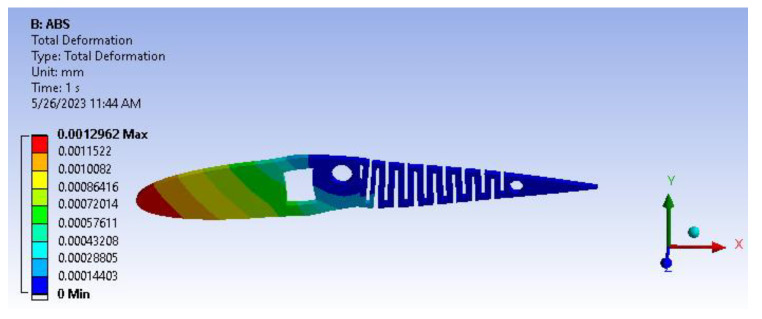
Total deformation.

Figure 13 illustrates the total deformation of the camber, which measures 1.296 µm. This value remains well within the design criteria limit of 2 µm, determined based on the total length of the span. Considering the ABS material, which exhibits brittle behavior under deformation, fatigue life analysis reveals that the areas with high-stress concentration are the square through hole slot, circular pockets and ribs. However, the resulting deformation for ABS amounts to only 1.2 µm, which is relatively low and will not significantly impact the aforementioned regions, even considering the presence of sharp corners. Nonetheless, as per design standards, these areas will be filleted or chamfered to enhance user comfort and maintenance. Introducing fillets or chamfers will further mitigate the stress concentration zones, thereby increasing the sustainability of the fatigue life cycle.

**Figure 14:**
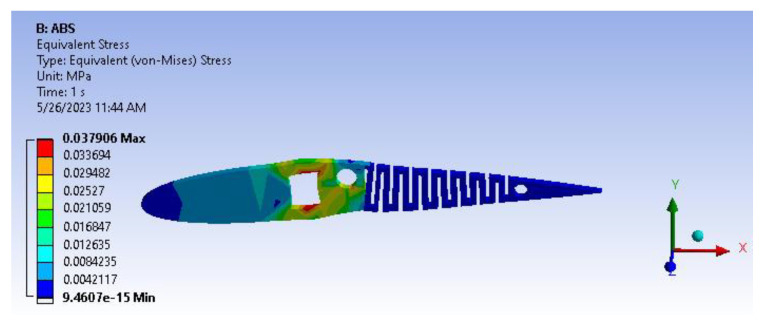
Von mises stress.

Based on the information provided in Figure 14, it can be deduced that the von Mises stress value is 0.037 MPa. This value is deemed negligible, indicating that the structure has the potential to endure beyond its intended design life cycle due to its current robustness.

**Figure 15:**
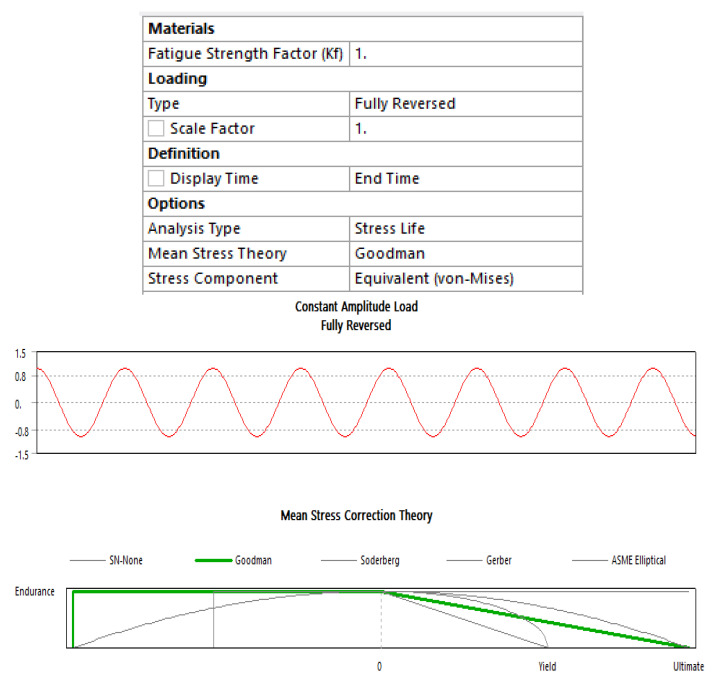
Fatigue input parameter details.

**Figure 16:**
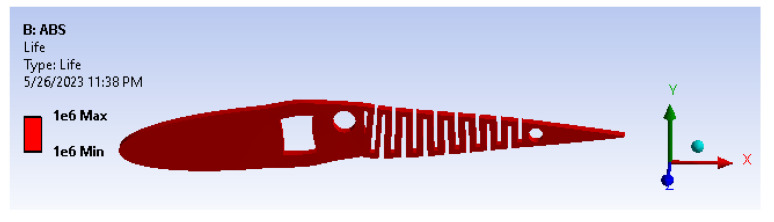
Fatigue life.

The structural analysis results, including total deformation and von Mises stress, have prompted further investigation into the fatigue behavior of the wing under the given load conditions. This study is depicted in Figure 15, where input parameters are determined for fully reversed conditions, and the mean stress theory is applied using a modified Goodman case with a stress-based approach. Figure 16 illustrates the results of the fatigue life analysis, indicating cases of infinite life cycles beyond 100,000 cycles. Specifically, the results demonstrate that the model can serve for more than 1,000,000 cycles, implying an infinite lifespan for the applied loads and boundary conditions based on the evaluated load cases. Moreover, considering the improved structural rigidity achieved by the presence of transverse bars connecting the cambers across the entire wing span, the fatigue life results remain acceptable when considering the worst-case load scenario on each individual camber. It is important to ensure that sharp corners are filleted or chamfered, as this further enhances the overall structural integrity of the wing.

**Figure 17:**
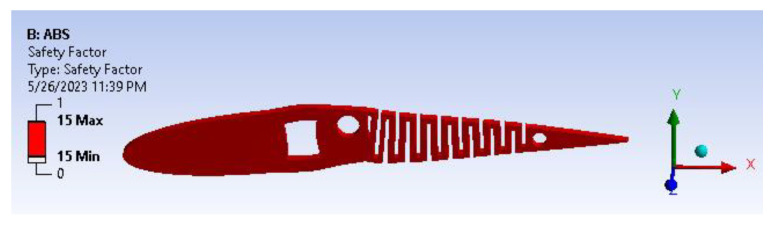
Fatigue factor of safety.

**Figure 18:**
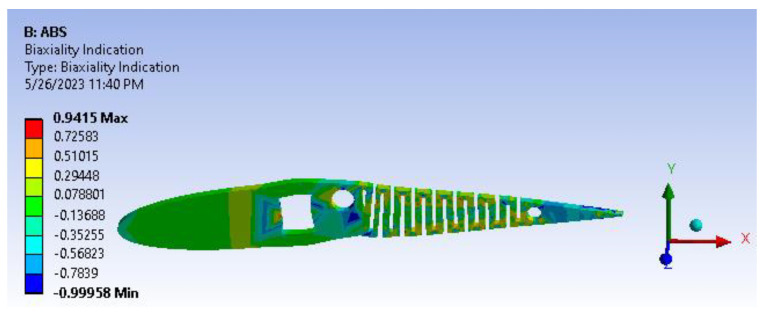
Fatigue biaxiality indication.

A factor of safety calculation showed the factor of safety ranging well within the desired/eligibility criteria of 1.1 to 1.5. As the image depicts in Figure 17, the factor of safety is more than three. Furthermore, there is significant scope for optimization and modifying the camber. From Figure 18, biaxiality indication details show tension and compression behavior based on the load pattern.

#### 5.4.2. PVC

**Figure 19:**
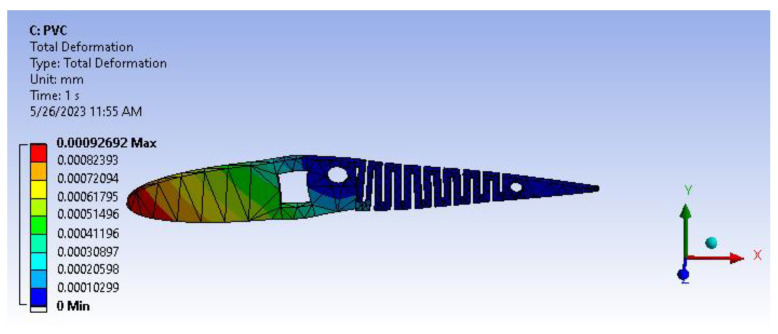
Total deformation.

**Figure 20:**
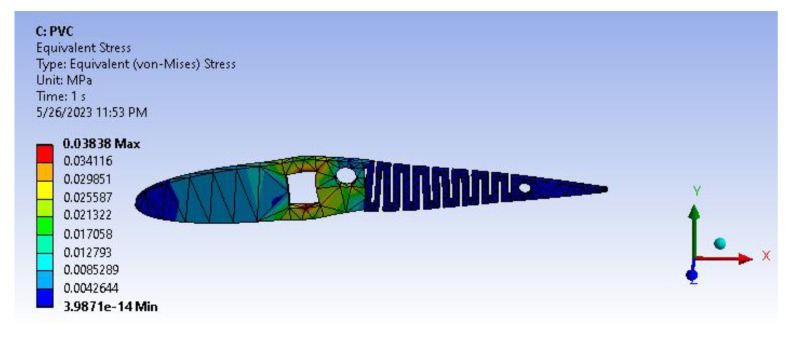
Von Mises stress.

PVC has been a recent inclusion in additive manufacturing, such as 3D printing systems, to diversify the application of PVC manufacturing. Figure 19 and Figure 20 show total deformation and the von Mises stress of the active camber.

**Figure 21:**
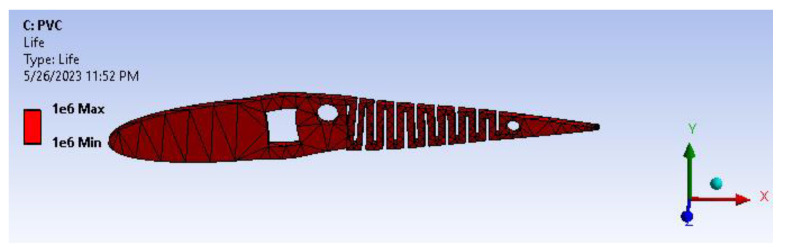
Fatigue life.

**Figure 22:**
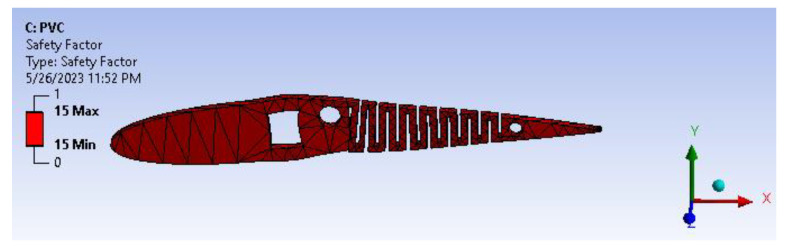
Factor of safety.

**Figure 23:**
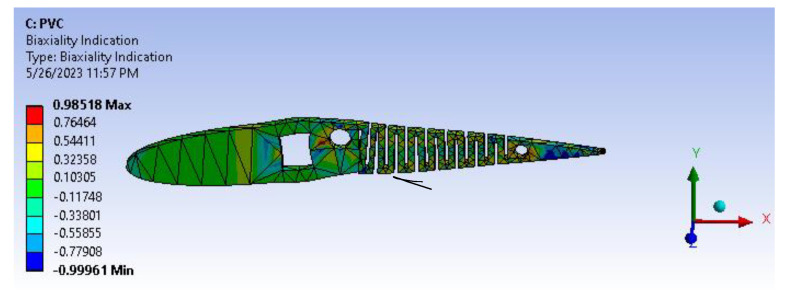
Biaxiality indication.

Figure 21 infers the fatigue life analysis showing infinite life cycle cases, which are beyond 100,000 cycles. The results show more than 1,000,000 cycle cases, meaning the model will serve an infinite life span for the applied loads and boundary conditions. A factor of safety calculation showed the factor of safety ranging well within the desired/eligibility criteria of 1.1 to 1.5. As the image depicts in Figure 22, the factor of safety is more than 3. Further, there is a lot of scope for optimization and modifying the camber. From Figure 23, biaxiality indication details show tension and compression behavior based on the load pattern as the PVC material has a slightly higher density of 1.25 gm/cc in comparison to ABS with 1.12 gm/cc; PVC has a higher correlation to real-time applications than ABS.

Since the material in consideration is PVC, which exhibits ductile behavior under deformation, a fatigue life analysis reveals that the areas with high-stress concentration are the square through hole slot, circular pockets and ribs. However, the resulting deformation for PVC amounts to only 0.9 µm, which is relatively low and will not significantly impact the aforementioned regions, even with sharp corners.

Nevertheless, in accordance with design standards, these areas will still be filleted or chamfered to enhance user comfort and facilitate maintenance. The introduction of fillets or chamfers will further mitigate the stress concentration zones, thereby increasing the fatigue life cycle sustainability of the wing.

## 6. Conclusions

This study introduces a novel concept for a morphing mechanism that incorporates a bio-inspired geometry and a structurally designed prototype using ABS material. The primary objective of this mechanism is to minimize drag and enhance lift during flight, thereby potentially reducing an aircraft’s fuel or power consumption. The outcomes obtained from this research encompass the following achievements:

1. This design is particularly suitable for short take-off and landing (STOL) aircraft and those operating in stall and low-speed flight conditions, providing better engineering and economic performance and improved functionality compared to conventional wing systems. It is well-known that real-world problems on Earth are nonlinear, and this is also true for the behavior of the skin. However, this study focuses solely on the aerodynamic approach using CFD and does not consider the structural analysis behavior. Although researchers are working on using man-made materials to address this issue, the cost aspects of such solutions remain difficult to estimate at this time.
2. In this case, the maximum displacement at 30 degrees of deflection is 47.45 mm, and the maximum stress is 21 MPa. The yield strength of ABS material is 41.14 MPa. This kerf morphing structure with a safety factor of 2.5 can withstand structural and aerodynamic loads.
3. Analysis results of flap and morph showed 27% efficiency, validated through convergence criteria in ANSYS CFX.
4. Furthermore, for future work, it is recommended to conduct comprehensive experimental studies accompanied by a detailed comparative analysis between analytical and experimental results. At the initial stage, it is crucial to prioritize simulations that incorporate all relevant parameters based on real-time conditions. This will enable a more thorough understanding and evaluation of the system under study.
5. The fatigue calculations based on the stress-based approach indicate that PVC material has a higher life-carrying capacity compared to ABS. This implies that PVC is more resistant to fatigue failure and can endure a greater number of load cycles before experiencing structural degradation.

## Figures and Tables

**Table 1:** Characteristic nature of curve (slope).

Slope	$C_{l_{\propto }}$	$C_{d_{\propto }}$	$C_{m_{\propto }}$
Morphed airfoil	5.0630	0.0326	−0.4868
Flapped airfoil	2.3412	0.0859	−0.3436

**Table 2:** Analysis result of flap and morph.

Description		Lift (N)	Drag (N)	Lift/Drag	Mean Lift/Drag	Efficiency (%)
MORPH	Ansys	2.89	0.47	6.20	5.74	27%
	Theoretical	2.86	0.49	5.80		
	Simflow	2.86	0.55	5.21		
FLAP	Ansys	2.39	0.49	4.88	4.50	
	Theoretical	2.37	0.52	4.57		
	Simflow	2.37	0.58	4.10		

## Data Availability

Not applicable.

## Abbreviations

α	Angle of attack (AOA)
Cl	Lift coefficient
Cd	Drag coefficient
Cl/Cd	Lift–drag coefficient ratio
Cm	Pitching movement coefficient
TE	Trailing edge
Cp	Centre of Pressure
$C_{l_{\propto }}$	Coefficient of change in lift with respect to change in angle of attack
$C_{d_{\propto }}$	Coefficient of change in drag with respect to change in angle of attack
$C_{m_{\propto }}$	Coefficient of change in moment with respect to change in angle of attack
L/D	Lift to drag
UAV	Unmanned aerial vehicle
NASA	National Aeronautics and Space Administration
DARPA	Defense Advanced Research Projects Agency
FishBAC	Fish bone active camber
MAC	Mean aerodynamic chord
NACA	National Advisory Committee for Aeronautics
ABS	Acrylonitrile butadiene styrene
CFD	Computational fluid dynamics
XFLR5	Analysis tool for airfoils, wings and planes operating at low Reynolds numbers
Pugh Chart	Method used to analyze different ideas and to determine the optimal choice
STOL	Short take-off and landing
